# New quantitative method for dental wear analysis of small mammals

**DOI:** 10.1038/s41598-022-26705-x

**Published:** 2022-12-23

**Authors:** Sandra Bañuls-Cardona, Ruth Blasco, Jordi Rosell, Anna Rufà, Josep Vallverdú, Florent Rivals

**Affiliations:** 1 Institut Català de Paleoecologia Humana i Evolució Social (IPHES), Edifici W3, Zona Educacional 4, Campus Sescelades, Universitat Rovira i Virgili, 43007 Tarragona, Spain; 2 Àrea de Prehistòria, Departament d’Història i Història de L’Art, Facultat de Lletres, Universitat Rovira i Virgili (URV), Avinguda de Catalunya 35, 43002 Tarragona, Spain; 3 Departament de Prehistòria i Arqueologia, Universitat de València, Avd. Blasco Ibañez, 28, E-46010 València, Spain; 4 ICArEHB-Interdsciplinay Center for Archaeology and the Evolution of Human Behaviour, Universidade do Algarve, Campus de Gambelas, 8005-139 Faro, Portugal; 5 Univ. Bordeaux, CNRS, MCC, PACEA, UMR 5199, 33600 Pessac, France; 6 ICREA, Pg. Lluís Companys 23, 08010 Barcelona, Spain

**Keywords:** Climate sciences, Environmental sciences

## Abstract

The application of dental wear study to murids has always been ruled out because of their omnivorous diet, which does not leave significant wear on the dentition. Nevertheless, in our work we select *Apodemus sylvaticus* (wood mouse) as the object of study for several reasons: its seasonal diet, its ability to resist the gastric juices of predators, the fact that it has not undergone major morphological changes since its appearance 3 million years ago, and its widespread distribution throughout much of Europe and part of Africa. The importance of this work lies in the modifications we make to the dental wear methodology for its application to murids. These enable us to obtain quantitative data on the entire tooth surface. The sample chosen was a total of 75 lower first molars from two different archaeological sites: Teixoneres cave and Xaragalls cave. The chronology of the samples chosen ranges from Marine Isotope Stages 5–3. The data obtained reveal that the part of the tooth that shows most wear is the distal part (entoconid). Furthermore, the results provide us with relevant information on the types of accumulations of remains in the caves (short vs. long term), as well as on the seasonality of Neanderthal occupations during the Upper Pleistocene (MIS5-3) of the northeastern Iberian Peninsula.

## Introduction

Dental l wear, a term coined by Fortelius and Solounias (2000), is a technique used to reconstruct the dietary traits of extinct species, allowing diet to be studied over a long timeframe. It is based on the changes in the occlusal relief and shape of the teeth that occur over an animal’s lifetime, or at least over a time scale long enough for seasonal diet changes not to be detected^1^. Thus, it reflects “the average diet of a particular species from a particular location in space and time”^2^.

The dental wear methodology has been applied to brachydont and hypsodont ungulates (e.g., Refs.^3–9^). In ungulates, hypsodonty has long been recognized as an adaptation for grazing: grazing is suggested to increase tooth wear due to the endogenous (e.g., fibre, silica) and/or exogenous (e.g., dust, grit) properties of ingested food^10–13^. However, it is unknown whether tooth crown height is correlated with the mastication of the high fibre or silica content of grasses, the ingestion of external abrasives, or both. Rodents have a shorter life and a more accelerated early life than other mammals. The grass-eating species are significantly more hypsodont than frugivorous or folivorous species, and arboreal rodents are less hypsodont than terrestrial species^14^. Within Rodentia, there are many hypsodont species (the Cricetidae family), but the Muridae family is omnivorous and its tooth morphology is bunodont, i.e., lacking precise occlusion and a permissive temporomandibular joint allowing extensive condylar displacements in three dimensions^15^ as found in pigs, primates, and humans. In Muridae, the upper molars have supplementary lingual cusps and are characterized by three longitudinal rows, whereas the lower molars still have two rows of cusps. In the upper and lower molars, crests connect cusps into transverse lophs with a characteristic V shape^16^. The molars are of limited growth, non-renewable, and with roots used to crush food.

In recent years, dental wear and microwear studies have been carried out on small mammals^17–24^, but the application of these methodologies to murids is less frequent than to other rodents^25–27^. The reason for this is that the study of dental wear in rodents was for years dismissed: first, because of their propalinal chewing (i.e., from back to front)^11^; and secondly, the case of species such as *Apodemus sylvaticus*, because of their omnivorous diet, which results in low abrasion and leaves less clear signs on the tooth.

In this paper we will try to overcome the limitations on the study of dental wear in rodents by the application of an innovative methodology. In this work we do not use the method of dental microwear, i.e. we do not analyze the micro-features of tooth enamel that reflect the diet of an individual in the last days of his or her life. Instead, we have studied the macroscopic tooth wear. We will analyse the European wood mouse, *Apodemus sylvaticus* (Linnaeus, 1758), a rodent belonging to the Muridae family. This species has been present in the Western Palearctic region for the past 3 Ma^28^. Moreover, we propose some changes to the dental wear methodology for its application to small-mammal fossils, concretely Murinae, the subfamily under study. This modified methodology will provide qualitative and quantitative information on murid tooth wear, shedding light on the changes in the diet of these rodents.

## Sites

**Figure 1:**
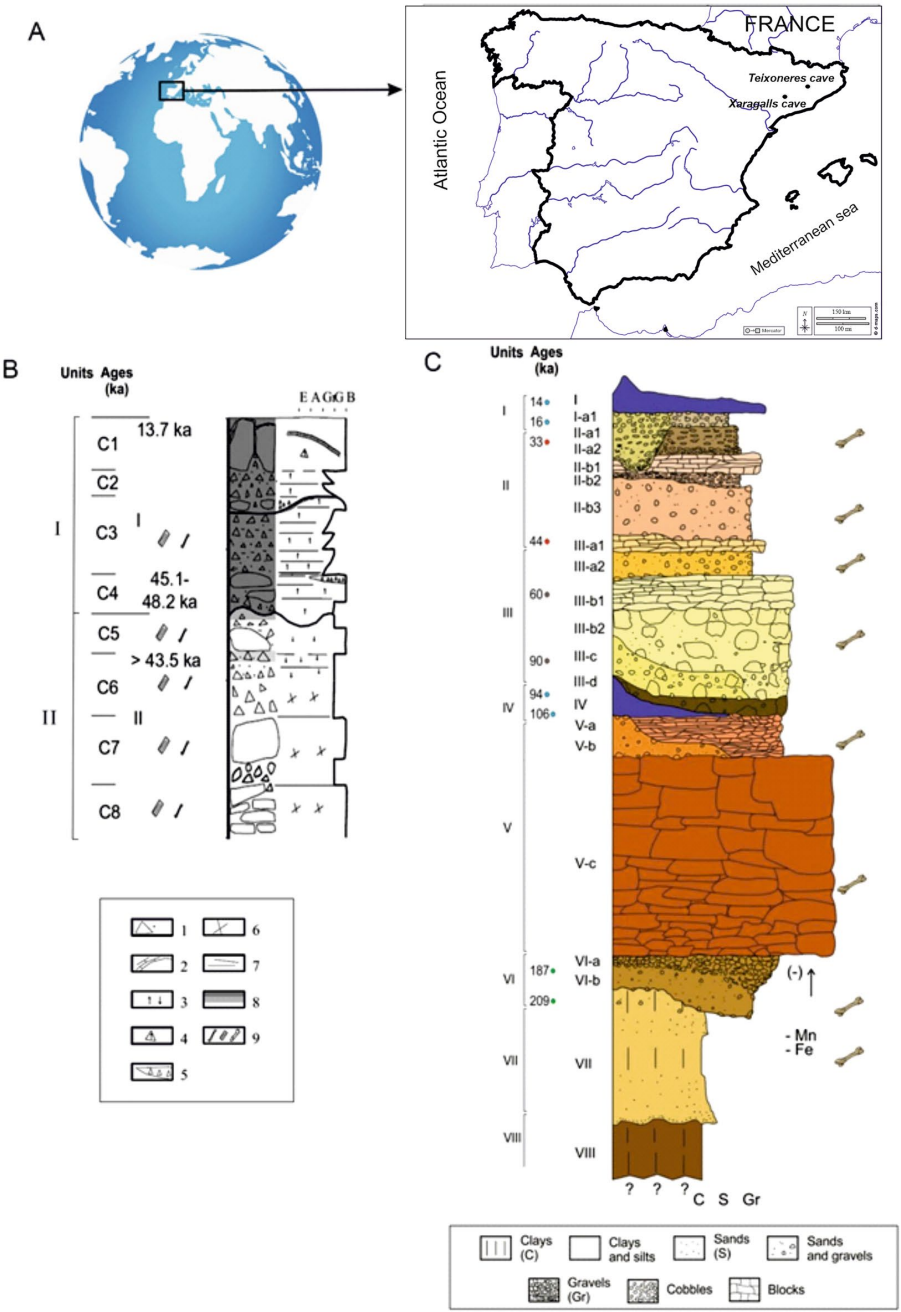
(**A**) Location on a Map drawn from an open image bank (https://d-maps.com) and stratigraphic information on (**B**) Xaragalls cave and (**C**) Teixoneres cave.

For the present paper, two sites are selected: Xaragalls cave and Teixoneres cave. These sites are located in the northeastern Iberian Peninsula and belong to the Upper Pleistocene (Fig. 1).

### Cova dels Xaragallls

Cova dels Xaragalls is a karstic system located in Vimbodí-Poblet (Tarragona, Spain). It lies on the left margin of Castellfollit Creek at the head of the Francolí River Basin, in the Poblet Forest at an altitude of 590 m a.s.l.

There are two main parts, the entrance (Holocene deposits) and the ‘Sala Gran’ gallery (Pleistocene deposits), at the end of the cave system. There are numerous studies of the entrance part^29–31^. These studies document pottery remains^30^ as well as human remains from burials within the cave^31^ and other elements that evidence Chalcolithic and Bronze Age occupations^29^. At ‘Sala Gran’, produced by the phreatic evolution of an extensional plane^32^, were found in 2008^33^ Pleistocene levels, divided in two units. Unit 1 is composed of gravel and blocks with clastic support that is poorly organized to stratified, and a structure filled with red sandy clays and fossil remains. This unit contains 4 layers, C1 to C4. In the layers C1 and C2, there are fossil remains. Layer C1 is a speleothem that covers blocks and semi-filled gravel. A sample from this speleothem has been dated by the U/Th method to 13,723 ± 99 years. *Pinus*-type *sylvestris* charcoal from layer C4 has been dated by ^14^C AMS to 45,120–48,240 cal. BP (UGAMS-8123). Unit 2 is composed of gravel, limestone rocks with clastic support, and lithostratigraphic units (C5–C8) with coarsening grains. An undetermined charcoal fragment from layer C6 has been dated, providing an age outside the range of the ^14^C method, which may mean that it is older than 43,500 years (Fig. 1)^34^.

### Cova de les Teixoneres

Teixoneres cave is located near Moià (Barcelona, Spain) at an altitude of 760 masl The cave is approximately 30 m long, lying within a karst system developed in Neogene limestone (Collsuspina Formation). It is composed of three different chambers (X, Y, and Z). It has two entrances corresponding to chamber X (main entrance) and chamber Z (smaller access).

The cave preserves an extensive stratigraphic sequence made up of eight units that show complexity in the occupation of the site. U-series dating was undertaken on samples from the stalagmite layers present in units I and IV, respectively overlying and underlying the levels studied here (Fig. 1). Sub-units IIa, IIb, IIIa, and IIIb range between 14 ky BP (unit I) and 100 ky BP (unit IV)^35^. The radiocarbon ages of animal bone remains, some of which were modified by humans, indicate that the human presence in Unit III spanned the period > 51,000–44,210 cal. BP while unit II dating extends between 44,210 and 33,060 cal BP^36^.

The biochronology of the rodent assemblages (characterized by the presence of *Pliomys lenki*, *Microtus* (*Iberomys*) *cabrerae*, and *Hystrix* sp.) limited the chronology of Units II and III to between ca 30 and 90 ka BP^37^. Moreover, the latter study reveals warm and humid conditions in Unit III that could be associated with an interstadial period in MIS 5a, whereas Unit II presents cold and dry conditions probably associated with a Heinrich event (H3–H5) in MIS 3^37–39^. Recently, Zilio et al.^40^ analysed the spatial distribution of the archaeological record of Unit III, formed during Marine Isotope Stage 3, with the aim of understanding the spatial organization and activities pursued by the Neanderthals and carnivores (bears, hyenas, as well as smaller carnivores). These authors confirmed the initial working hypothesis of Rosell et al.^41,42^, which was that the site was occupied by Neanderthal groups in a similarly structured and consistent way throughout the entire time range covered by subunits III-a1, III-a2, and III-b, and that this favoured the creation of a spatial pattern dominated by fire-use related zones in the outer area of the cave. At the same time, the presence of carnivores is clearly indicated in the archaeological record, although these did not result in significant spatial perturbations of the site.

## Material and methods

### Sample

The small-mammal assemblages studied in this paper pertain to Xaragalls cave (Unit 1) and Teixoneres cave (Units IIIa and IIIb). These samples were identified and published by López-García et al.^33^ and López-García et al.^37^. In Xaragalls, the *Apodemus sylvaticus* remains comprised 402 specimens and 147 individuals, whereas in Teixoneres there were 116 specimens corresponding to 58 individuals.

The most recent dental wear studies of Muridae have used the upper molar M1 and the lower molar m1^26^. In our case, we focused on the first lower molar (m1) of the wood mouse; concretely, at least 10 individuals (preferably 20) should be scored to adequately sample the variation within the population. We selected 25 first lower molars from Xaragalls Unit 1, 25 from Teixoneres subunit IIIa, and 25 from subunit IIIb. These selected molars present five main tubercles, the anterolingual and anterolabial tubercles (tF, tE, tD, tC) confluent in X shape. Acording to Pasquier (1974), these are the representative morphology of *A.sylvaticus*.

### Remarks on the taphonomy

The taphonomic study of a microvertebrate accumulation is very important for several reasons. Firstly, as with other fossil material, it is important to know the type of accumulation in question. Normally, every fossil undergoes chemical changes in the biological environment and the diagenetic environment after the death and burial of the animal. In the case of small mammals, moreover, it should be borne in mind that, in most cases, they are preyed upon by birds or carnivores, and this produces changes in the morphology of the bones and teeth^43^. In the present context, a taphonomic study determining the agents responsible for the rodent accumulation at both sites has already been carried out and published^33,37^. Taphonomic observations of the Teixoneres assemblage indicate that the predatory agent was a category 1 avian predator (sensu^44^) such as the barn owl (*Tyto alba*), an opportunistic predator that produces slight modifications in the bones of its prey. At the Xaragalls site, an owl (*Athene noctua*) is responsible for the accumulation. This small nocturnal raptor is also considered an opportunist^45^ and produces moderate modifications in its prey^44^. The rodent in question, *Apodemus sylvaticus* (wood mouse), has teeth that are very resistant to the effects of gastric juices^46^, so although the predators are characterized by moderate digestion, their effects should not hamper the study of the dental wear.

In addition, breakage is often caused by tooth wear, resulting in irregular surfaces that lack the smooth polish typical of well-preserved enamel^7^, so teeth without breakage should be studied to avoid any bias in the study.

### Morphometric analysis

Biometric data of the first lower molars (m1) were obtained using the measurement method established by Pasquier (1974), which involves measuring the length and width of the tooth. These measurements are of the tooth in occlusal view, at its maximum length and at its maximum width. The length (L) of m1 was measured as the maximum distance between the mesial and distal part of the tooth. The width (W) was determined as the projection on a straight line perpendicular to the axis of length between the most distant parts of the lingual and labial sides of the tooth.

In this study, measurements were taken using the image treatment software *ImageJ* on photographs taken with the *Hirox KH-8700 3D Digital Microscope* (dual illumination revolver zoom lens MXG-5000REZ and triple objective turret with objectives ranging from 35 to 5000 × magnification). This software allows one to obtain actual measurements based on photographs, which in this case correspond to the occlusal plane of the studied teeth.

### Dental wear methodology

Green and Croft^7^ argue that, since dental wear is scored at a gross level with the naked eye, it is only necessary for the development of the tooth facets to be observed. Fortelius and Solounias^2^, however, argued that a dental wear analysis of an ungulate mammal requires two qualitative variables of a tooth to be taken into consideration: the occlusal relief (height of the cusp), and the shape of the cusp (scored as sharp, rounded, or blunt). Subsequently, a single-variable method for scoring dental wear was proposed^4,47^. This method combined occlusal relief and cusp height into a single qualitative (categorical) score ranging from 0 (high relief and sharp cusps) to 3 (blunt and essentially unrelieved cusps). The authors based their method on the observation that occlusal relief and cusp sharpness are correlated with each other. This modified version was named mesowear II^48^.

Ulbricht et al.^26^ analysed the dental wear of murine rodents using the mesowear II method with the same variables as in ungulates, revealing high occlusal relief, blunt cusps, and sharp cusps. The individual molar cusp shape and relief scores were converted into a single dental wear score as follows: a combination of high relief and sharp cusps was assigned a score of 0; a combination of high relief and rounded cusps was assigned a score of 1; a combination of low relief and rounded cusps was assigned a score of 2; a combination of low relief and sharp cusps was assigned a score of 2.5; a combination of low relief and blunt cusps was assigned a score of 3.

**Figure 2:**
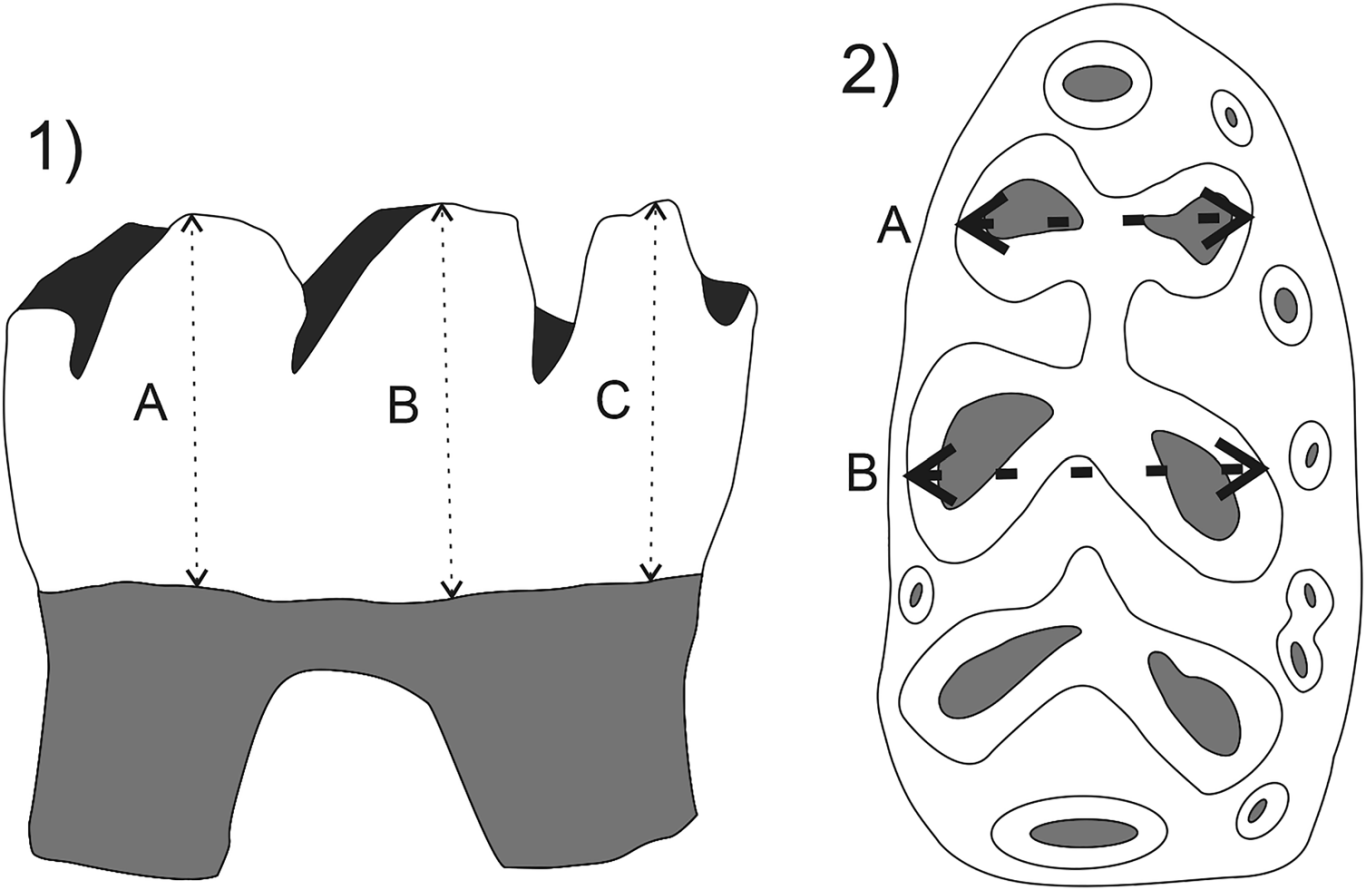
Mesowear methodology. (1) Measurements of the molar crown of the first lower molar: (**A**) anteroconid; (**B**) metaconid; (**C**) entoconid. (2) Measurements of the molar crown of the first lower molar: (**A**) anteroconid from labial to lingual part; (**B**) from metaconid to protoconid.

In this paper, we analyse the relief using the *Hirox KH-8700 3D Digital Microscope* (dual illumination revolver zoom lens MXG-5000REZ and triple objective turret with objectives ranging from 35 to 5000 × magnification). From the images obtained we measured the dental wear using *ImageJ*, an image processing software. The choice of tooth position and wear facet to sample needs to be optimal to have a standardized sampling protocol that targets the same wear facet on the same tooth across all individuals^49^. Concretely, we measured the height of m1 from the tartar line to the highest point of the crown, at three points in labial view. From the mesial to the distal part of the first lower molar in labial view, these were: 1, entoconid; 2, metaconid; 3, anteroconid (Fig. 2). Moreover, we found that the dental wear produced changes in the morphology in occlusal view. For this reason, we measured: 1, the anteroconid from its labial to its lingual part; 2, the distance from metaconid to protoconid (Fig. 2).

### Statistical data analysis

The first step was to report the descriptive statistics (mean and median, among others). The next step was to find out whether our sample represents a normal distribution, for which we used the Shapiro–Wilk test^50^.

Assuming a normal distribution, we applied the most widely used parametric test for independent data belonging to more than two groups, ANOVA^51^, specifically Levene's test. We used this test in accordance with Rivals et al.^1^, and we used univariate statistical tests to compare means and examine correlations using Pearson's coefficient^52^. These distributions were visualized using box plots and violin plots as methods for plotting numerical data. The violin plot is like a box plot, with the addition of the probability density of the data at different values, usually smoothed by a kernel density estimator.

## Results

### Biometric data

The measurement method established by Pasquier^16^ showed there to be first lower molars of different sizes in the sites under study (Length = L and Width = W). However, these differences are statistically "normal" according to the Shapiro–Wilk test. The small mammals teeth accumulated in Teixoneres IIIb (TxIIIb) are the longest in length, whereas the greatest width was recorded in Xaragalls cave. However, the overall values of all the samples, according to Levene's test, are close to each other, which indicates a very low difference among them. In the case of the width, the values vary a little more, and some significant differences are observed.

Analysis of the kernel density estimator of the length indicates that the Xaragalls (Xa) sample has a greater concentration of larger individuals, whereas the sample from Teixoneres IIIa (TxIIIa) shows the smallest size, and sample TxIIIb is a more heterogeneous group in terms of size.

### Dental wear: labial view

**Figure 3:**
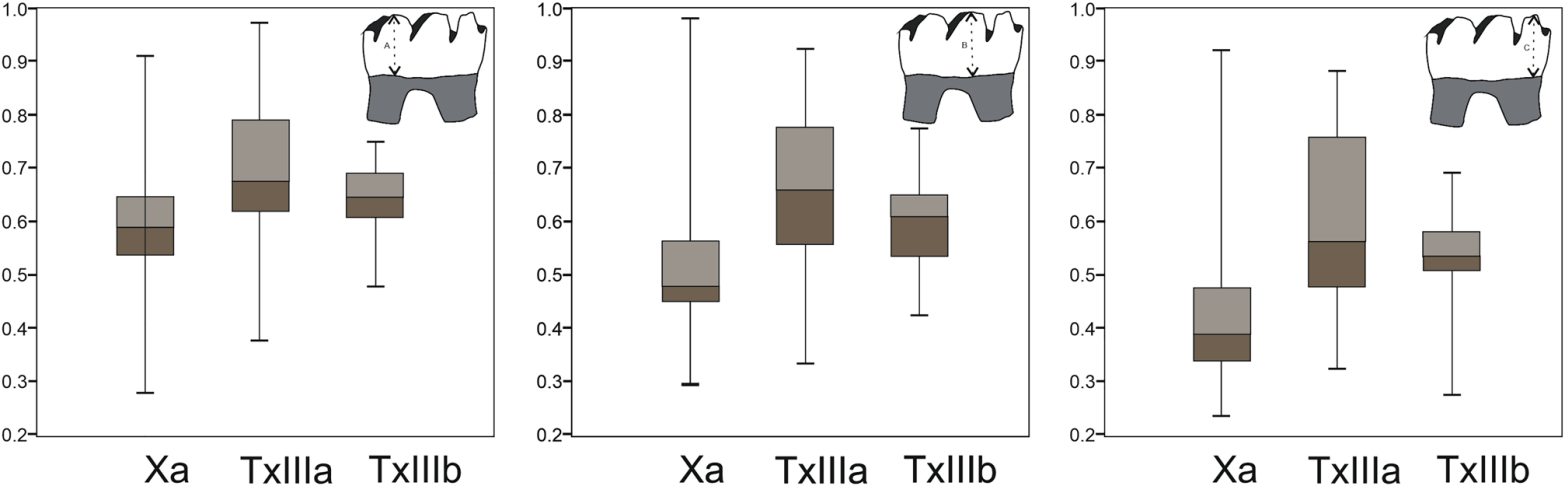
Boxplots of labial mesowear results: (**A**) anteroconid, (**B**) metaconid, (**C**) entoconid. Samples: Xa from Xaragalls cave; TxIIIa and TxIIIb from Teixoneres cave. Measurements in millimetres.

The data obtained from the analysis of dental wear in lateral view suggest the existence of two groups. One of these groups contains samples Xa and TxIIIb and is characterized in general by very homogeneous wear in both cases, except for a few specimens in Xa. The other group consists of sample TxIIIa, whose wear is highly heterogeneous (Fig. 3).

For a more detailed analysis of the teeth belonging to all the samples studied in this work, we compared the average wear of the mesial part of the teeth (anteroconid) using Levene's test. The values obtained in general show significant differences, whereas if we analyse each sample separately with the Shapiro–Wilk test, we see homogeneous wear in each of them, albeit with a small difference in the sample from subunit IIIa.

Applying Levene’s test to the measurements taken in the middle part of the molars (metaconid) from the three samples indicates that there are no significant overall differences between them. However, the study of each of them separately shows small differences, as in the case of sample IIIb.

Finally, the analysis of the distal part of the tooth (entoconid) indicates some differences with respect to the mesial (anteroconid) and middle part of the tooth (metaconid). There can be seen no significant differences in wear in this area between all the teeth analysed (75 molars) according to Levene's test. However, an examination of each sample separately shows some differences among individuals. Applying the Shapiro–Wilk test revealed that the distributions are normal, whereas small differences are observed in TxIIIb. In this sample the attrition shows greater variability; i.e., there is a lower number of individuals with wear data around the mean. Moreover, sample TxIIIb shows the lowest dental wear value of the three samples.

It can be said in general that the wear is located in the distal part of the tooth, or entoconid, in all cases. The highest value of labial wear in entoconid part is recorded in Xa, and TxIIIb shows in this part of the teeth the most homogenous dental wear values (i.e., low variability).

### Dental wear: occlusal view

A statistical analysis of the occlusal wear measurements using Levene’s test shows there to be no significant differences among them. The data are distributed within the range of "normality" according to the Shapiro–Wilk test. However, a detailed analysis of the mesial and middle part of the teeth indicates some small differences.

**Figure 4:**
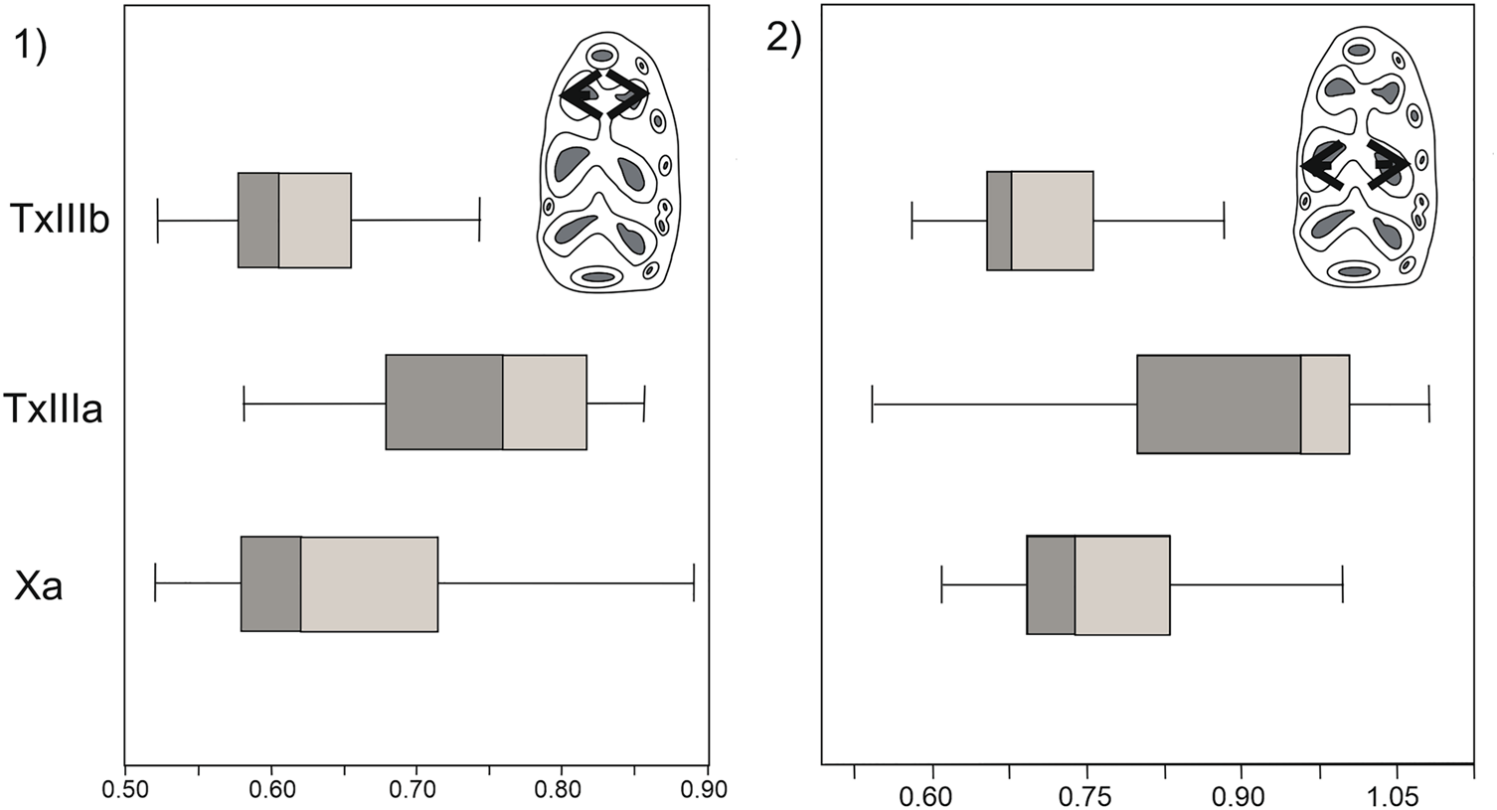
Boxplots of occlusal mesowear results: (1) anteroconid, (2) metaconid-protoconid. Samples: Xa from Xaragalls cave; TxIIIa and TxIIIb from Teixoneres cave. Measurements in millimetres.

In the case of samples Xa and TxIIIa, a lower concentration around the mean is observed in the mesial part of the teeth, whereas sample TxIIIb shows a lower concentration around the mean in the middle part of the tooth (Fig. 4).

Regarding the occlusal part of the teeth under study, it can in general be said that no wear pattern can be established. Furthermore, the data obtained for lateral and occlusal wear were analysed using Pearson’s coefficient, and no correlation was found between the two measurements. In the case of the two samples from Teixoneres, indeed, they are almost completely independent.

## Discussion

Dental wear patterns are helpful in reconstructing palaeoenvironmental conditions, but open questions remain about seasonal variations in diet^26^. In our case, the generalist but highly seasonal diet^52,53^, the ability of murids to learn and develop new feeding strategies in a short time, and individual variables such as rearing condition, sex, and weight had no effect on the feeding behaviour of *Apodemus sylvaticus*^54^. This allows us to make some preliminary comments to shed light on these questions.

### Dental wear and the palaeoenvironment

**Figure 5:**
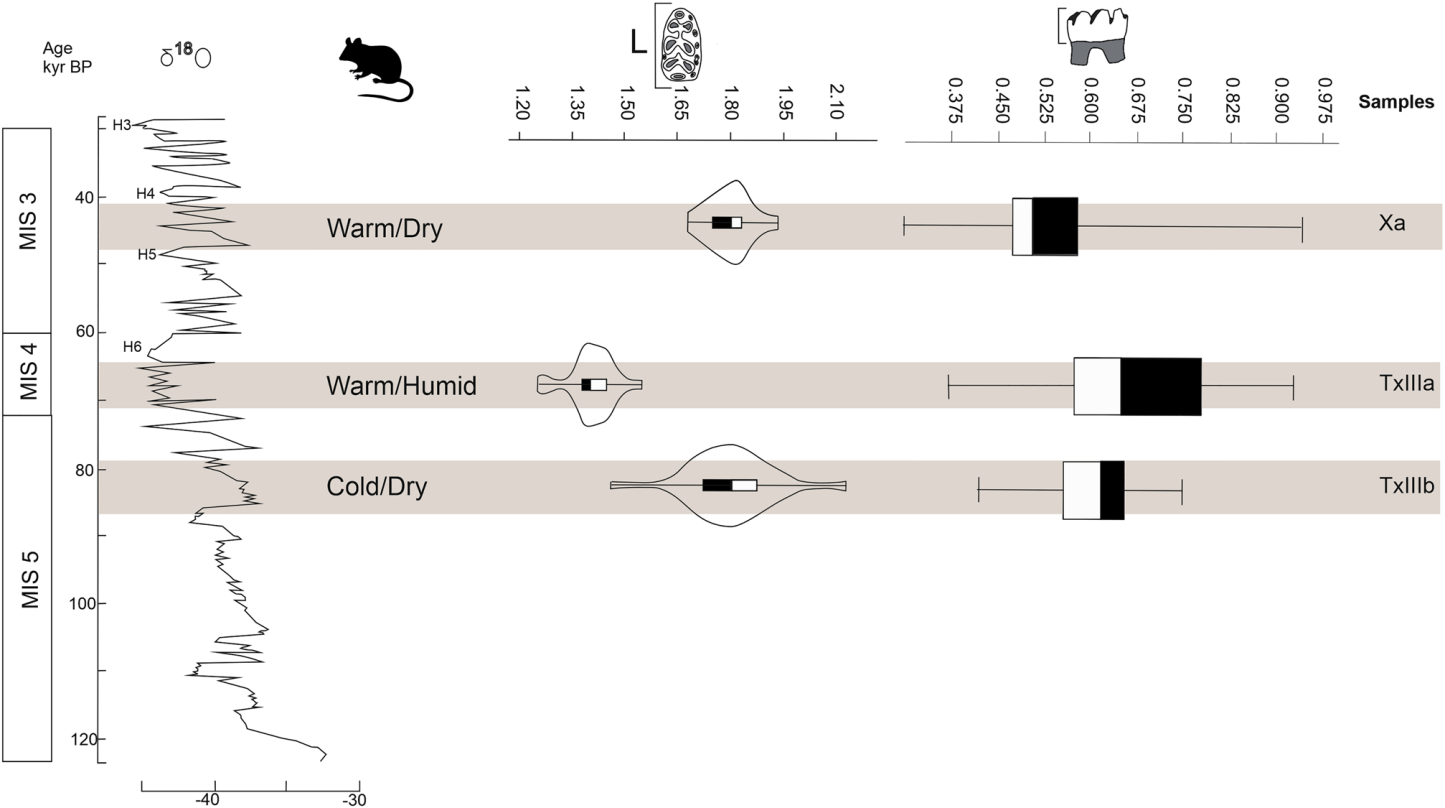
Synthetic results obtained from the mean results of the labial mesowear analysis on boxplot and length data of the molars on violin plot with a kernel density estimator. All of these are contrasted with the ^18^O-isotope curve (modified from Sánchez-Goñi and d’Errico^8^), and with palaeoenvironmental and palaeoclimatic data obtained from small vertebrates^33,37^. The grey bars indicate the samples.

The three samples analysed correspond to different chronological periods, specifically to three different isotopic stages (MIS 3, 4, and 5), and each of these stages has different climatic characteristics. The oldest sample is the TxIIIb sample, which falls chronologically within MIS 5 (ca. 128–74 ky BP). This period was characterized by an initial mild phase called the Eemian or MIS 5e (ca. 128–110 ky BP), which saw maximum expansion of the Mediterranean forest in southwestern Iberia. This phase was followed by four cold periods alternating with four mild periods (ca. 110–74 ky BP), characterized by the development of steppe formations in cold intervals, and the expansion of open forest in warm intervals^55–57^. The next oldest sample, TxIIIa, falls within MIS 4 (ca. 74–60 ky BP). MIS 4 was characterized by summer insolation minima over the northern latitudes of the northern hemisphere, producing maximum extension of ice caps, a sea level hundreds of metres below the current level, and low ocean temperatures^56^. Finally, the sample analysed from the Cova dels Xaragalls (Xa) is part of MIS 3 (ca. 60–30 ky BP), characterized by major rapid climatic changes showing high variability (Fig. 5). The pollen record from MIS 3 reveals a dynamic whereby forest development and semi-desert expansions alternate, linked with warming and cooling phases of the sea surface, respectively^58,59^.

Even though the sites belong to very different chronologies and marine isotope stages, palaeoenvironmental analyses based on the small vertebrates indicate a predominance of wooded and shrubby areas, amounting to more than 70% of the total in all cases^33,37^. This habitat is very favourable for the development of *Apodemus sylvaticus*. Currently, although it is a fairly generalist species and its habitat range varies during the seasons, it chooses areas of dense shrub or tree cover^60^. During the Pleistocene too, it is considered to have been a thermophilic, forest species, as it seems to have been more abundant in relatively mild climatic conditions, when it was associated with other species in wooded environments. By contrast, it was scarcer in the colder phases, disappearing from the most northern areas of Europe during some of the phases of the Last Glacial Maximum^28,61–63^. Given the palaeoenvironmental interpretations of the small-vertebrate associations of these sites, the food resources should not differ greatly among the three samples. However, through the study of dental wear, differences can be observed. There are significant differences between the samples in both the lateral plane and the occlusal plane. Samples Xa and TxIIIb can in general be seen to present more similar wear, with most of the individuals presenting levels very close to the average. However, analysis of the mean dental wear values reveals small differences among samples that could be related to climatic differences^33,37^. In TxIIIb (MIS 5), colder and drier conditions are recorded, whereas the values obtained for the dental wear indicate low abrasion. In Xa (MIS 3), by contrast, the climatic conditions are also dry but warmer, and the dental wear data point to very marked wear, and in TxIIIa, the conditions are warm and humid, and the dental wear data are indicative of the lowest abrasion in the sample. We thus observe that the difference that exists between them could be a result of the environmental humidity (Fig. 5). This could be for two different but interrelated reasons: food and birth rate.

### Dental wear and dietary traits

Given the ethology of *Apodemus sylvaticus* at present, its demographic dynamics shows strong seasonal variations that mainly depend on changes in environmental conditions and the abundance of acorns^64^. The prevalence of this rodent is also observed to decrease during the summer period in the Mediterranean area^65,66^, and in the northeastern Iberian Peninsula summer drought is even thought to be the main cause of *Apodemus* mortality^61^. Older bibliographic data on the reproduction of field mice in the Iberian Peninsula^67,68^ indicate that the sexual activity of the species takes place preferably from spring to autumn, whereas more recent data point more towards summer reproduction, especially in the Mediterranean region^52^. However, other authors^53,69^ have argued that it is not possible to delimit some periods of sexual activity and others of sexual rest for the species. In short, it can be said that various biotic factors, including the amount of food available, climatic conditions, and local meteorological phenomena, have a strong influence on reproduction. It could be that suggests that the humid conditions recorded in TxIIIa favoured a greater variety of foods, and a greater quantity of foods such as fleshy fruits, mushrooms, shoots, etc.^52,53^ that are known to cause lower dental abrasion than seeds, another favourite food of *Apodemus sylvaticus*. It should also be noted that the teeth analysed from this level are considerably smaller than those from samples TxIIIb and Xa. In light of the previously cited theory that benign environmental and trophic conditions would have favoured greater reproduction, this small size could be attributed to a younger population. In determining the relative age of specimens, Felten^70^ argued that with age these teeth undergo wear and tear, which is often used to differentiate juveniles from adults. This subject has sparked some discussion, because although it is true that the wear of the molars is related to the age of the individual, the diet of the individual should not be ruled out as a factor. In the present case, however, the smaller size of the teeth in the sample could also be due to humidity, since according to the study of *Microtus arvalis* by Luzzi et al.^71^, the size of the teeth increases in times of greater aridity. This hypothesis should therefore be taken into consideration in the case of *Apodemus sylvaticus*.

A more detailed analysis of the teeth reveals more pronounced wear in the mesial part of the tooth (m1), specifically in the distal part of the tooth (entoconid). This contrasts with other studies such as the one by Ulbricht et al.^26^, where the dental crowns present a significant specific relief observed in the distal part. Although murines chew in a propalinal direction^72^, this could indicate that the observed differences in wear might be related to the seasonal feeding of *A. sylvaticus* rather than the type of chewing.

Despite this general trend in wear, there are some patterns to be considered in the samples analysed. The highest values for lateral wear are documented in Xaragalls and IIIb from Teixoneres, with Xaragalls showing the most worn teeth. The study of the lateral view of the teeth from Xaragalls shows that in 76% of the sample the wear does not exceed 0.5 cm in the distal part of the tooth or entoconid. The mesial part of the tooth (anteroconid) and the middle part (metaconid) show greater relief with very homogeneous values. The study of the relief in occlusal view indicates a greater opening of the lobes in the middle part of the tooth, corresponding with the values established in lateral view. All these data indicate that the accumulation from Xaragalls (Xa) is likely to have occurred in late autumn and early winter. It is in this period when a more abrasive diet composed of fruits and seeds is documented in these small rodents^50^. Taphonomic data support this same period of accumulation. The taphonomic study of this sample revealed that *Athene noctua* was responsible for the accumulation^33^. This predator has seasonal feeding patterns. Its staple food is insects and small mammals, but this may be supplemented by amphibians and small birds during spring and summer, related to the breeding season of this species^73^, whereas during the early winter period its main prey is *Apodemus sylvaticus*^74^.

In general, we assume that the most worn part in all the samples analysed is the distal part of the tooth, specifically the entoconid. However, analysis of the data from the mesial and middle part reveals differences between the samples studied, as indicated by Levene’s test. In the case of the Teixoneres samples, the values obtained from the study of all the variables indicate greater wear in sample TxIIIb. All the individuals analysed in this sample show very homogeneous wear; i.e., their wear is very close to the average, which means that the members of this group were subject to the same dietary characteristics throughout their short lives, with foods of high abrasive power, in the case of *Apodemus* a diet rich in seeds. These characteristics indicate that the accumulation occurred at a cold time, probably in winter. There are several reasons why the remains accumulated in winter. One of these reasons is that since the rodents have a life expectancy of one year, and their maximum reproduction period is autumn, it is quite likely that the time of their death was when they emerged from their winter torpor. This species does not hibernate, but it can go into torpor if low temperatures lead to food deprivation^49^. Another point to be borne in mind in the case of Teixoneres is that the taphonomic study shows the accumulator in both levels to be a category 1 predator, probably *Tyto alba*^37^. During the winter, the barn owl focuses its diet on murids, which make up almost 50% of the biomass consumed, whereas the summer diet is more varied^42^. This very specific winter diet explains the homogeneity of the group. Finally, this hypothesis contrasts with the palaeoenvironmental interpretation of this same level using different proxies, according to which the climatic conditions were characterized by low temperatures and a certain environmental aridity^37,75^.

### Dental wear and Neanderthal occupations

Finally, this study of dental wear in rodents also helps us to study the settlement patterns of the Neanderthal groups. In most cases, archaeologists only focus on periods when sites have been occupied by humans. Previous studies have made it possible to characterize the Neanderthal settlements at Teixoneres as a succession of short-term occupations^39,75^. In our case, the accumulations of small mammals occur at times when the cave was not occupied by Neanderthals, so the information provided by the dental wear pertains to the period between the human occupation of level IIIb and that of IIIa. This was a short-lived event since the dental wear observed is very homogeneous. The foods consumed led to a high level of wear and were probably mostly fruits and seeds. The occupation between IIIa and IIb, by contrast, was more prolonged, with more varied wear, meaning that the specimens in question consumed a wider variety of foods.

## Conclusions

The conclusions drawn from the present work are broadly twofold, pertaining to the methodology and to the data obtained.

On a methodological level, it can be seen how the modifications made to the dental wear technique for its application to murids provide quantitative data that allow us to detect differences in dietary patterns which had not been established until now. This should be further developed in future publications. It has also been shown that dental wear is a method not only for determining the age of individuals, but also establishing that, as in other rodent families, humidity is an important factor in the size of individuals.

On the other hand, the palaeoenvironmental data obtained have been contrasted with other proxies, showing this methodology to provide data of great palaeoenvironmental and climatic importance. It also helps us to ascertain types of human occupation and the duration of phases without human occupation.

## Supplementary Information

Supplementary Information.

## Data Availability

All data generated during this study are presented herein and included in the Supplementary Information.
